# Turning the Co-Production Corner: Methodological Reflections from an Action Research Project to Promote LGBT Inclusion in Care Homes for Older People

**DOI:** 10.3390/ijerph15040695

**Published:** 2018-04-07

**Authors:** Paul Willis, Kathryn Almack, Trish Hafford-Letchfield, Paul Simpson, Barbara Billings, Naresh Mall

**Affiliations:** 1 School for Policy Studies, University of Bristol, 8 Priory Road, Clifton BS8 1TZ, UK; 2 School of Health and Social Work, University of Hertfordshire, Hatfield, Hertfordshire AL10 9AB, UK; k.almack@herts.ac.uk; 3 Department of Mental Health, Social Work, Interprofessional Learning and Integrative Medicine, School of Health and Education Middlesex University, Ground Floor, Town Hall Annex, The Burroughs, Hendon, London NW4 4BT, UK; p.hafford-letchfield@mdx.ac.uk (T.H.-L.); bbillings@gmx.com (B.B.); anamsoul@yahoo.co.uk (N.M.); 4 Department of Applied Health and Social Care, Edge Hill University, St Helens Road, Ormskirk, Lancs L39 4QP, UK; simpsonp@edgehill.ac.uk

**Keywords:** care home policy and practice, co-produced research, older LGBT people, care home residents, resilience, risk environment and change

## Abstract

*Background*: Older lesbian, gay, bisexual and trans (LGBT) residents are often invisible in long-term care settings. This article presents findings from a community-based action research project, which attempted to address this invisibility through co-produced research with LGBT community members. *Particular Question*: What conditions enable co-produced research to emerge in long-term residential care settings for older people? *Aims of Project*: To analyse outcomes and challenges of action-oriented, co-produced research in the given context. In particular, we explore how co-production as a collaborative approach to action-orientated research can emerge during the research/fieldwork process; and reflect critically on the ethics and effectiveness of this approach in advancing inclusion in context. *Methods*: The project was implemented across six residential care homes in England. Reflections are based on qualitative evaluation data gathered pre- and post-project, which includes 37 interviews with care home staff, managers and community advisors (two of whom are co-authors)*. Results and Conclusions*: We discuss how the co-production turn emerged during research and evaluate how the politics of this approach helped advance inclusion—itself crucial to well-being. We argue for the value of co-produced research in instigating organizational change in older people’s care environments and of non-didactic storytelling in LGBT awareness-raising amongst staff.

## 1. Introduction

The social inclusion of diverse groups of older people in long-term care settings is a prominent topic on the current health and social care agenda, as evident in current UK policy pushes to implement equality standards into social care practice [1]. This is an important area to address given that the number of older people requiring care and support in later life is predicted to rise and demands for housing with health and social care provision are likewise expected to increase [2]. At present, we know very little about what constitutes optimal conditions for promoting inclusion practices in residential care homes and how residents and staff in these settings can foster meaningful, inclusive relationships. Older lesbian, gay, bisexual and transgender (LGBT) adults are one marginalised group of residents whose lives and identities are too-often invisible or disregarded in these environments [3,4].

While some of the issues facing LGBT residents in care homes are likely to be similar for all older people, research studies into the lives of older LGBT people identify issues unique to the lifecourse of this social cohort that care home staff need to consider if seeking to provide holistic and person-centered care [5,6,7,8,9,10,11,12]. Older LGBT people live with a legacy of times when social attitudes were less tolerant and legislation criminalised rather than protected LGBT citizens [8,13,14,15]. Consequently, some older LGBT individuals have maintained privacy about their sexual orientation and/or gender identity over a lifetime or experience a renewed reluctance to disclose central aspects of their identity in order to feel safe when accessing health, social care and housing provision in old age [11,13,16,17,18,19]. We also know that in comparison to heterosexual adults 50+ years, older LGBT people have a greater likelihood of living alone, are less likely to have children to call upon for support and are therefore more likely to look to friendship networks for support or rely more heavily on formal care services in later life [18,19]. For older LGBT individuals, moving into a care home can be experienced as a “double trauma” given the need to adapt to communal living [3] coupled with anxieties about being obliged to conceal differences in gender identity and sexual orientation [15].

In this paper we outline the methodological journey of a community-based action research project, which attempted to address the invisibility of older LGBT adults in residential care homes through co-produced research with LGBT community members. To our knowledge, there is little exploration of co-produced research with LGBT people that addresses sexual and gender-based inequality in the provision of social care and welfare services, the work of Fannin et al. [20] and Kong [21] being exceptions to this. However, this methodology can enhance research practice with socially marginalised groups of older people, including LGBT service users. Co-produced research provides an innovative platform for producing knowledge (or “findings”) in collaboration with people who are potential future recipients of care home services and who share both a personal and political investment in addressing concerns about social exclusion, unequal treatment and marginalization in the delivery of services. At the heart of co-produced research is the promotion of partnership working across both the design and execution of research and in doing so the avoidance of replicating unequal relationships and the re-marginalisation of historically invisible groups such as older LGBT adults. 

The project was implemented in 2016 across six residential care homes in a large city in England and encouraged care home managers and staff to address the inclusion of older LGBT-identifying residents with the support and input of community volunteers. Participating care homes provided residential care for older people with a range of needs, some with older residents living with complex physical disabilities and dementia. Community advisors (CAs) were volunteers recruited from the local community in which the project was based and were all individuals who had some experience and understanding of social care services based on previous work and life-experiences. All identified as LGBT except for one volunteer who identified as an “LGBT ally” and all participated in the same training. CAs were recruited because of their explicit commitment to enhancing the social inclusion of older LGBT people in social care and housing services. The main findings and outcomes from the project have been reported elsewhere [22].

This article has two aims: (1) to explore how co-production as a democratising approach to action-orientated research can emerge during the research and fieldwork process; (2) to reflect on the efficacy and ethical challenges of this approach for advancing a social inclusion agenda in care home settings for older people. For this methodological discussion, our overarching question is: ‘What conditions enable co-produced research to emerge in long-term residential care settings for older people?’ We conclude that there are various enabling conditions that supported what we describe as a “co-production turn” and examine what conditions facilitated this change in direction. Substantially, this involves identifying ways in which the research shifted from a linear, top-down process of gathering information to a transformational, bottom-up initiative that was anchored in creating empathic spaces for mutual sharing of experiences, views and beliefs within care home services. We show that researcher reflexivity and commitment to community engagement can prompt emergence of co-production during the fieldwork process. Before embarking on analysis, we review current practices concerning co-produced research with user and community groups. 

## 2. Co-Produced Research with User/Community Groups: What Is Known?

In the wider context of service delivery and development, co-production is defined as “working in partnership by sharing power with people using services, carers, families and citizens” [23] (unnumbered page). Within the research literature it is difficult to pinpoint a shared definition or definitive method for approaching co-produced research. However, its commonality lies in the shared objective of “unsettling traditional relations between expert and public knowledge” [24] (p. 145) and disrupting the more conventional power asymmetry between researcher and those researched. As a methodology, this approach to research provides a democratising platform for the inclusion of multiple parties involved in the production of knowledge (university researchers, user/participant groups, community organizations, for example) [25,26].

Co-production approaches to research are increasingly expected across a range of disciplines. In health and social care, these approaches are an extension of policy directives promoting personalisation of care and self-directed support [23]. Co-produced research represents an extension of participatory action research and feminist approaches to research, which seek to democratise research processes and address power asymmetries [27,28]. Co-produced research is closely allied with “transformative research” [29], which has relevance for marginalised groups such as LGBT groups and communities seeking to redress patterns of discrimination and marginalisation for LGBT citizens. In a critical piece concerning co-production projects, Kara [26] usefully illuminates tensions arising over multiple identities, indicating multidimensional power asymmetries, which in turn, can thwart democratic and egalitarian working practices. Vennick et al. [30] have also highlighted the potential rhetorical nature of co-production within health care settings, in which project teams need to reflect critically on the consequences and desirability of adaptations and tailoring actions when carrying out quality improvement projects. 

In general terms, co-production can help challenge power asymmetries whilst adding depth and breadth to academics’ and community members’ understandings of their cultures [31]. Hardy et al. [31] have shown how co-production approaches can minimize health inequalities when they are implemented from the outset, invest in participants (training and support), and select participants through experience and potential (not only via qualifications). Equally, in his participatory action research with older gay men in Hong Kong, Kong [21] has demonstrated how the emergence of a collaborative approach during the research process can transform the researched to researcher in the shift “from hierarchy to collaborative partnership” (p. 9). 

Further, Beebeejaun et al. [32] outline four key aspects of the broader ethos of participatory, co-productive research, namely developing more equal partnerships with communities and practitioners; fostering mutual learning and interaction to understand issues; maintaining this ethos throughout to ensure some forms of knowledge (for example, academic knowledge) are not privileged over others; and, working to implement findings for transformational change. However, they also acknowledge significant challenges to this ideal including funding deadlines; conflict and discrepancy between researchers’, community members’ and other stakeholders’ knowledge interests and priorities; inherent power relationships between the different stakeholders; and, adherence to institutional rules on research governance. 

In practice, co-produced research is not just about immersion of researchers in participants’ accounts and experiences (itself a power asymmetry) but also a shift towards collaboration [21]. It seeks to move from establishing dialogue and partnership between researchers and researched towards managing and overcoming hierarchies between them to produce knowledge collaboratively that can change attitudes and practices [33]. Co-production poses challenges concerning the negotiation of boundaries, power dynamics and ground rules between social researchers and partner communities [32,33]. This requires a shift for researchers from the traditional stance of producing findings and recommendations (“fixers of problems”) to becoming facilitators who find solutions through collaborative efforts with service users, underpinned by principles of reciprocity and mutuality. It can also involve, in the interests of equality, consciously suppressing the researcher voice (thus avoiding the trap of the omniscient author who faithfully reflects “objective” truths about participant experiences) to highlight participant-researcher voices [21,24,34]. Kara [26] also highlights the necessity of reflexive ownership of (inter-)subjectivity and positioning as ways of generating credible and improved knowledge.

The above-identified practices can also help to position service users/participants as experts on their own circumstances. As Bell and Pahl note, this requires constant attention to shifting relations of power and domination across a two-way knowledge flow [35]. Also, researchers can lose sight of the fact that they have to traverse boundaries between academia and their own membership of minority communities [36]. The academic authors of this paper span the boundaries between activism and working in voluntary and statutory sectors across social care while representing differing points of identification with LGBT identities.

## 3. Methods and Materials: Introducing the Two-Stage Project and Its Evaluation

The overarching aim of the project was to pilot and evaluate a scheme within six care homes in a large city in England that would enhance the inclusion of older LGBT residents. Achieving this goal required attention to several related concerns as follows: (1) providing opportunities for care staff to develop awareness and knowledge of working with older LGBT people; (2) enabling care home managers to demonstrate leadership in an area of equality and diversity and to receive support in shaping the culture of their own care home environment; and (3) a requirement for an evidence-based assessment and development tool to “audit” and benchmark good practice in LGBT inclusion. 

The project focused on social inclusion across the two social dimensions of sexual identity and gender identity. This is an important distinction to make as gender identity is often disregarded in research on LGBT people, leading to experiences of trans-identifying people being overlooked in LGBT-focused research [37]. More specifically, gender identity encompasses one’s internal sense of gendered self and how individuals express or present their gender to others [38]. Other authors use the term “gender self-designation” [39] to capture how individuals designate their own gender identity, which can differ from the “genders typically associated with their externally assigned ‘sex’ classification” (p. 160).

The project comprised two phases. The first phase involved an action research collaboration with an independent housing provider and six of its care homes for older people, the project leader and the eight CAs. It was also supported by the provider’s LGBT advisory group. The second phase involved an evaluation led by an academic research team, which commenced three months after the first phase had begun. While involved in the planning and design of the project as ‘insiders’, this team was not involved in the first phase. 

### 3.1. Phase One (January–August 2016): Implementing the Scheme

Eight CAs were recruited to the project. In terms of age profile, four CAs were 65 years; two were aged between 55–64 years; one CA was aged between 45–54 years and another between 35–44 years. Two CAs identified as lesbian, three as gay, one as ‘queer’ (indicating sexual and/or gender fluidity) and one as transgender. CAs were from White British, Jewish, British-Asian, and Bangladeshi backgrounds. They were recruited using the participating organization’s volunteer process, which included references and criminal record checks as safeguarding measures. 

The intended role of CAs was to spend concentrated periods of time in allocated care homes and complete a broad audit and assessment exercise over a four-month period to ascertain the degree of social inclusion of LGBT people within these homes. Volunteers received a small honorarium payment and coverage of expenses. Following an initial training programme, CAs were allocated in pairs to work with participating homes. The participating homes were sampled pragmatically given time and budgetary constraints. CAs’ role involved talking to residents, staff and other stakeholders about the inclusion of LGBT residents and viewing internal policies on equality and other key documents. The academic team devised an assessment and development tool to inform CAs’ discussions with staff and residents, to promote conversations about care home processes and interactions within homes, and as a means of recording CAs’ observations [40]. This tool was based on good practice guidelines concerning older people, LGBT inclusion and equal treatment in health and social care services [41,42,43], and was structured around seven domains: policies and procedures; environment; consultation with community groups; risk management (including responding to discriminatory language and abuse); issues specific to gender/trans support and care; cultural safety; and, end-of-life care planning. At the end of phase one, CAs met with managers, along with the project leader, to share concerns and to identify ways of building on good practice. A ten-point action plan was formulated that captured feedback from CAs and managers and agreed actions for managers beyond the life of the project. Care home champions were appointed in each participating home to drive forward agreed actions from inside the organisation. 

### 3.2. Phase Two (March–October 2016): Evaluating the Scheme

The second phase, commencing three months into the project, comprised a qualitative evaluation of the project led by the academic team. Ethical approval was granted on 7 March 2016 by the Faculty of Medicine and Health Sciences Ethics Committee, University of Nottingham (affiliation of author Almack at the time). Semi-structured telephone interviews were conducted by the team before intervention (to generate a sense of participants’ hopes and concerns for the project) and three to four months afterwards (post-intervention) to establish actual experience of the project. A total of 37 interviews were conducted with 19 respondents:

1 Pre- and post-intervention interviews with CAs (*n* = 8) and care home managers (CH) (*n* = 8). These semi-structured telephone interviews (lasting between 30 and 40 min) aimed to capture CAs’ and managers’ feedback on the initial intervention, challenges encountered and perceived outcomes.
2 Interviews with key informants (*n* = 3), which included semi-structured interviews with the Community Engagement Officer and an external facilitator who led the initial CA training session.

In addition, notes and transcripts from four meetings between CAs and managers were included as additional data-sources. Below, we present insights from our reflections on the research process as the academic team alongside in-depth reflections from two co-authors, Barbara and Naresh, who participated in the project as CAs. Barbara and Naresh were CAs first then co-authors second. As co-authors, both individuals have been actively involved in the dissemination of findings from the project to wider audiences and both were keen to contribute to this paper. We locate the reflections of CAs alongside our reflections as academic researchers and in doing so move away from privileging the voices and views of the academic team only. We also draw on interview material from pre- and post-intervention interviews with care home managers and CAs and share some interview quotes to help illustrate our reflections and to present a wider range of views. 

## 4. Reflections on the Journey: Telling the Co-Production Story

### 4.1. First Encounters and Initial Resistance 

Here we discuss CAs’ introduction to the project aims and their first encounters with managers of participating care homes (see Box 1). The initial training sessions were intended to help CAs to “find their direction” on the project, to introduce them to the assessment and development tool and establish rapport between them.

Box 1 **Reflections from CAs.**

**Barbara (lesbian):** *At the start of the project, I felt a bit lost, out of my depth, a lesbian on the fringes of politics amongst a group of politically aware fellow community advisors. I felt we all grappled to find a clear direction and to get the project off the ground. This was turned around by strong supportive leadership from (project leader), training from (external consultant), enthusiasm from care staff in (participating company) and the goodwill of everyone involved. During the initial training, I was challenged by my lack of LGBT history awareness and my lack of understanding of transgender and intersex issues. All were sharp learning curves helped by the training provided and shared knowledge of colleagues...*

The above commentary indicates how some CAs were initially feeling “out of their depth” or challenged by their perception of having insufficient knowledge about the topic. These concerns were addressed partly in the training sessions. The sessions enabled CAs to learn about each other’s life-experiences, skills and knowledge and, in doing so, gaps in their understanding of different life-histories and experiences across the LGBT identity spectrum. This process was enhanced by group sessions with the project leader who provided emotional and practical support throughout phase one.

The assessment tool represented unfamiliar territory and some CAs hinted at reservations about how and when to use the tool:

**CA training feedback**: *I think it may be useful to go through the toolkit in sizable chunks and follow up with practical exercises. But, I do feel that once CAs meet and build relationships with CH managers and further understand the dynamics of the CH and their residents, applying the toolkit will be easier.*

Early on in the process, CAs raised important concerns about the need for more rudimentary conversations with care staff and managers about individual beliefs, values and human rights as a necessary precursor to implementing the assessment tool. CAs also expressed concern about the highly structured format of the tool and how it could interrupt the flow of interaction between themselves, care staff and residents. This was a steep learning curve for the academic team. In retrospect, a more authentic co-production approach would have involved CAs in the development of such a tool, if, indeed, there was agreement about the need for a tool. 

Building initial relationships with participating care homes also proved challenging for CAs as they reported difficulties in making initial contact with managers with phone calls not being returned. This was also evident in their first group meeting with managers (March 2016) where there was low attendance by the latter. Telephone and email communication was often one-way and CAs sometimes felt they were being quietly “fobbed off”. Nevertheless, such feelings were counterbalanced by their recognition of pressures on managers:

**Interview comment from CA**: *It was more about getting managers along to meetings and getting them to engage with the volunteers ... Meetings were arranged but managers were too busy doing something else on the day... None of us has run a care home... It must be a perennial problem that something happens that you have to react to... For instance, there may have been several admissions on one day.*

These early challenges provoked a rethink of the project design as explained below. 

### 4.2. Rethinking Our Steps: Introducing Advisory Sessions in Homes

At a second CA group meeting (third month) with the project leader, it became clear that CAs were experiencing numerous challenges building relationships with care home staff. Indeed, some staff were considered lacking in understanding of LGBT issues to the extent that they were unable to respond to questions in the assessment tool. It appeared that more fundamental conversations were required. These concerns resonated with managers’ comments acknowledging low levels of awareness amongst care staff and service users of the identities and life-stories of older LGBT people. This observation by CAs marked a critical turning point where the project transitioned from being led by the project team to being co-led with CAs.

The CA group requested permission to set aside the tool to invest instead in co-facilitating further brief advisory sessions. These sessions were intended to initiate discussion with care staff and their managers about LGBT inclusion and prepare them more gently for questions and discussion embedded in the assessment process. This step-change required the academic team to trust in the experience and early insights of CAs who advocated a less structured, more inductive process that would allow care staff and managers to raise issues “upwards” through small-group conversations and awareness-raising exercises. As members of the academic team we had to suspend our reservations about putting aside (temporarily) an assessment tool that was central to the research process. Here, we concur with the view of Hardy et al. [31] who note that the “unexpected structures” (p. 593) or aspects of a project can be the most disruptive to egalitarian relationships, though we experienced such disruption as the basis of productive reconfiguration of power relations and the project itself. 

Accordingly, the project leader worked with CAs in small-group meetings to plan and develop the content and structure of advisory sessions. Content included: addressing language and terminology and explaining LGBT historical references; providing knowledge of current legislation relating to the rights, equality and changes for LGBT people in the UK; working through care home-based case studies to identify issues and strategies for action; and flagging up activities for promoting LGBT inclusion. Sample exercises had been modelled to CAs during initial training. Bringing further innovation into the process, CAs shared their stories of “coming out” to others about their LGBT identities, experiencing discrimination, and living as LGBT to help generate empathy from care staff. 

While implementing advisory sessions, CAs continued to engage in conversations with a range of stakeholders in parallel with the advisory sessions, which also infrequently included some residents and external visitors to care homes. 

### 4.3. Taking the Co-Production Turn: A Rocky Journey 

Barbara and Naresh’s reflections above touch on various obstacles (see Box 2). CAs encountered incidences which encouraged re-living of painful experiences, for example of being mis-gendered and/or discriminated against. LGBT people will have experiences of stigma, marginalization or discrimination and perhaps become adept at avoiding such encounters where possible. Despite the resilience of CAs, such accounts highlight ethical issues in co-productive research concerning the need to protect the well-being of research partners. At this point, the project leader was called upon to provide more intensive support to CAs.

Box 2 **Reflections from CAs.**

**Naresh (gay man):** *The project itself became a process of training carers and the organization in some of the issues facing the people from LBGT backgrounds to enable the carers to provide support and feel confident in service provision appropriate to their needs. I think there were narratives of exclusion evident either in the process or in discussions with some carers—seeing older people as being asexual (without any sexual identity) or “we don’t have any of those people here” rendering people from diverse gender and sexual identities invisible. In addition, heterosexual people hold expectations of what a gay, lesbian, trans person looks like and behaves. If individuals don’t “conform” to these stereotypes, then individuals like trans and bisexual people can be rendered invisible. Some residents may not meet carers’ stereotyped beliefs or be relegated to biologically gender congruent roles or attributed a heterosexual identity or cisgender. I was empathising with service users and how they may be being perceived and treated and how unsafe they felt and would feel being isolated.*

*There was some of the inevitable resistance to change and to being challenged within the organization. Challenging relationships with co-facilitators and also with the management at times felt like a re- experience of earlier trauma from growing up with confusion.*

**Barbara (lesbian):** *The strength of the negative misconceptions held by many staff about the LGBT community came as a complete surprise and was the most challenging to deal with. They found it hard to understand the needs of transgender and intersex people and sex reared its head as the main focus of care staff’s negative attitudes towards same-sex couples. In some cases, this was fuelled by their religious beliefs and culture. It was evident that they could not see the whole person, sexual activity was at the forefront and for some, fear that they may be ‘fancied’ by a member of the LGBT community.*

Implementing change was not without its difficulties, as identified across interviews with both CAs and managers. From the start of the advisory sessions, CAs reported challenges that tested their resolve. The first challenge (a practical-organizational one) concerned difficulties in ensuring the majority of care staff in each home attended group sessions. This proved challenging in the wider context of the daily demands of care home life and the constant ebb-and-flow of employees across shift patterns. Some CAs were resigned to the fact that they could not reach all staff members despite best efforts:

**Interview comment from CA**: *We couldn’t meet with all staff together formally. We quickly realised that, that didn’t work because all the staff were otherwise engaged… There were some people who didn’t take part. There was an admin worker who I thought actively avoided it—she was late, some work crisis or sick.*

By the end of the project, CAs had developed positive working relationships with managers who became instrumental in encouraging staff members to attend. However, this was not an easy point to reach. Occasionally, the support of managers would wane, which made it extremely difficult to organise meetings with care staff. In some instances, managers encouraged their staff members to attend sessions but did not attend themselves, which raised questions about the importance of leadership and modelling project engagement. 

Another practical-organizational challenge was the short-term nature of the project. The four-month time limit placed additional pressures on CAs to initiate sensitive and contentious conversations in a rapid time-frame that was not necessarily conducive to rapport-building. Some CAs expressed concerns that their advisory sessions were too brief to explore diverse issues concerning trans and intersex identities/issues, which are commonly overlooked, occluded by or even wrongly subsumed under the “LGBT” abbreviation.

A third set of more discursive challenges involved expressions of overt and covert resistance by care staff. CAs shared their observations of how some care staff members avoided discussions about LGBT lives or verbally expressed distressing, dehumanising views to CAs that were founded on stereotypes or fundamentalist religious beliefs. CAs described such conversations as a form of confrontation given that their own sexual or gender difference (and identity) was being framed as problematic, pathological (i.e., as a “disease”) or immoral. For example, one staff member expressed to the group concern about gay and bisexual male residents’ assumed predilection for sexual activity with animals or pets. Consequently, advisory sessions could be anxiety-provoking for CAs, especially when needing to challenge directly some staff members’ deep-seated beliefs:

**Interview comment from CA**: *One woman [staff member] stated she would ban her son from the house if he came out as gay but had worked quite happily with trans people. Some people were supportive, other people less so because of their religion and culture.*

These kinds of oppressive comments are difficult to challenge, as they strike at the heart of the social identification and community affiliations of LGBT individuals. It is unsurprising that care staff originating from societies in which sexual and gender difference are outlawed and/or attract severe moral condemnation will have generated hostility and unease. In 2017, 72 nation states were recorded as criminalizing same-sex sexual relationships, with 8 states (sovereign and regional) permitting the death penalty [44]. In parallel, hate crime and discrimination on the basis of sexual orientation and/or gender identity remains a contemporary problem for LGBT individuals in the UK, despite the legal protections available to LGBT citizens [45]. “In the moment” these morally-charged conversations tested the relationship between CAs and care staff:

**Interview comment from CA**: *One thing I didn’t anticipate was how much this experience touched on (my) painful memories of homophobia. For me, it was about being mindful of that personal impact. Looking after myself... The project leader is a massive support, really. You can speak to her about most things.*

These testing moments are suggestive of vicarious trauma in which CAs could feel reminded of anti-LGBT expressions encountered earlier in life. Facilitating training sessions in pairs enabled CAs to provide support to each other during and after each session. The project leader also provided ongoing support in person and over the phone throughout advisory sessions. While this level of support was not originally anticipated, it soon became vital to the welfare and resilience of CAs and the project’s longevity and chances of success. 

CAs recalled more covert statements from care staff that made LGBT identities invisible. Some staff members claimed not to have any LGBT residents in their care home and showed a lack of recognition of cues indicating sexual and/or gender difference. One manager stated in an interview:

**Interview comment from care home manager:***“Even if something existed out there, because there are no lesbian or gay customers, participation is likely to be very low”*

Statements like this presented a considerable hurdle for CAs who also had to contend with staff members’ presumptions of heterosexuality (i.e., assuming all residents are heterosexual until declared otherwise). These assumptions had to be questioned and unpacked before engaging in deeper conversations with staff. 

Despite the above-identified challenges, CAs persevered with leading advisory sessions and in some homes repeated session with different staff groups. Their perseverance paid off as CAs described “light-bulb” moments:

**Interview comment from CA**: *…there were huge benefits because there were some genuine conversations and movement. At the very best, there were some genuine light bulb moments and, most encouraging of all, people who held some entrenched views, have said, “I think differently now.”*

The above quote provides insight into the value of experienced CAs whose emotional and political resources facilitated their resilience in the face of ingrained hostility, as well as skilled negotiation around sensitive subjects. It also demonstrates awareness of the project’s significance in its potential to bring about and embed change towards reducing disparities in the delivery of health and social care services that is inclusive of LGBT service users. CAs were struck by the willingness of care staff to engage in difficult conversations and their honesty in naming their personal beliefs that may be in conflict with promoting LGBT-affirmative environments. Table 1 illustrates some of the key stepping stones during this process.

### 4.4. Taking Stock of the Outcomes 

While it is difficult to pinpoint substantial shifts in staff members’ attitudes and views, interview responses from both CAs and managers indicated that changes had taken place during the project. The advisory sessions were pivotal in enabling staff members to talk openly and explore with others, including LGBT individuals, the assumptions and wider social and biographical influences on their thinking and practice (see Box 3):

**Interview comment from CA**: *Most memorable moment? I think it was actually seeing a genuine shift from some of those workers and giving them the opportunity to say what they are struggling with. Getting people to think about people in terms of identity and about the fears of being marginalised. Seeing people shift a bit. That’s really good to see... Trying to appeal to people’s compassion.*

Box 3 **Reflections from CAs.**

**Barbara (lesbian):** *Adopting a person-centered approach to our advisory sessions and being willing to expose our own lives took staff beyond these thoughts to see that sex is not our driving force, but that family, friends, culture, sport etc.* *contribute to the whole person, and, yes, we are sexual beings as well. This was one of the most positive outcomes of the project as care staff shared that the advisory sessions expanded and changed their thinking towards the LGBT community in a positive way, opening their eyes to how their negative attitudes could oppress service users.*

*At the end of the project, I take away a sense of hope and belief that most staff are essentially caring people who want to provide a positive experience for the people in their care and, that with learning opportunities, change is possible, that negative attitudes towards the LGBT community can be challenged and changed, awareness gained, and an inclusive environment created. But, the project was not a one-off remedy for the six homes involved. The learning has just begun and needs to be nurtured and built on. Identifying LGBT champions in each home provided a platform for issues to be discussed and praise and encouragement given to support initiatives and ideas put forward by staff. This needs to continue to sustain positive change.*

**Naresh (gay man):** *I think the project established that the organization had a programme of training and recruitment to move them forward and an aim to provide more inclusive practice. The organization had shifted their commitment from a top-down to bottom-up approach. Any real and longer-standing changes in the adoption of more inclusive practice required a rolling programme of recruitment and training which explicitly addresses the issues for inclusion of people from LBGT communities. Change is difficult both on an individual and organizational level. Organizations need top-down and bottom-up approaches to change, as the managers need to buy into the project and have a clear direction.*

In the quote above, it appears that the advisory sessions were crucial in mobilising compassionate and empathetic responses from those with religious (or other) reservations to see LGBT individuals as expressing a form of difference (identity) as a personal right. Advisory sessions also appear to have deepened participating staff members’ understandings of the personal and social consequences of exclusion and marginalization on the basis of sexual orientation and gender identity. Taking a co-production turn in the project enabled these micro-discussions to occur between CAs and small groups of staff. 

Some CAs reported that the conversations that most successfully promoted attitude-change were those that involved CAs sharing their own stories of identifying as LGBT:

**Interview comment from CA**: *We (CAs) shared our stories with the groups—about coming out in later life. Talked about being baby boomers. That sort of created conversation... I gave my story to a care staff member (Asian woman) and she burst into tears.*

This account of storytelling highlights the value of non-didactic, experiential forms of awareness-raising that encourage empathy. Similar outcomes were noted by managers who, towards the end of the experience, were overwhelmingly positive about the influences of CAs and the role they had played in raising awareness and inspiring confidence:

**Interview comment from care home manager**: *One member of staff came up to me after the training. She’d been really resistant. In fact, she didn’t want to attend the training but afterwards she said it had really changed her whole perception.*

While it remains to be seen whether these outcomes translate into a long-term programme of change across participating homes, the feedback above suggests that the advisory sessions encouraged critical thinking—the power to think beyond, and possibly question, heteronormative logic (beliefs and assumptions that heterosexuality is the dominant social marker of sexuality and gender relations [46]). Shifts in attitudes were also attributed to collective actions within homes that would not have previously occurred. For example, one manager reported in an interview that her staff team had requested observing a two-minute silence after the Orlando gay nightclub killings in 2016. Other care homes introduced the wearing of rainbow ribbons by staff and managers alike to signal LGBT-pride and to initiate discussion with residents about the meaning of this symbol. 

## 5. Discussion: Reflections on the Co-Production Turn

Various points of learning arose from the risks and opportunities that evolved during the research. While the academic team did not begin with a co-production approach, the project took such a turn due to the CAs’ insights and reflexivity as they encountered discriminatory attitudes and views within the care home environments. It would be more accurate to describe the project as leaning towards co-production through more instrumental (though no less important) moments of collaboration with CAs. In this sense, we did not achieve “genuine co-production” as discussed by Beebeejaun et al. [32]. For example, the company’s LGBT advisory group was consulted on the content of the funding proposal and project outline, however, the bid was led by the academic team. We also acknowledge that the evaluation process was academic-led, partly to satisfy funding requirements, and that the scale of the study for initiating change was restricted to six residential care homes. 

Nonetheless, a co-production turn can be valuable in producing new, multi-faceted knowledge and understandings that reflect lived experiences and enhance the depth, credibility and authenticity of research findings. Making this turn strengthened our research approach in two critical ways. First, this approach enabled CAs, and in turn the academic team, to ascertain a deeper understanding of staff members’ and managers’ views and beliefs towards LGBT sexualities that we doubt would have been gleaned to the same depth through using more conventional, deductive approaches to data gathering such as the planned assessment and development tool. Second, the advisory sessions and storytelling method used within these sessions instigated a degree of individual and organizational change that would have been far less discernible without this form of structured interaction and personal exchange with staff and managers. Through a co-production turn, the project was able to recognise, value and make best use of different types of expertise, in this context the expertise of volunteer community members. It has also illuminated the value of using non-didactic awareness-raising methods that engage rather than alienate staff and of creating safer, non-judgmental spaces for critical exploration of long-held views reinforced over time (and over which non-LGBT individuals had little control). 

But, what mechanisms allowed the co-production turn to emerge? Certainly, the new direction proposed by CAs at an early point was pivotal in shifting the project from its pre-defined, structured approach for assessing care home environments to an inductive, dialogical approach re-oriented towards initiating change with care staff and managers through advisory sessions. As in Kong’s work [21], taking this risk shifted the research from a linear, top-down process of gathering information to a more transformational, bottom-up project that was change-led and anchored in creating empathic spaces for the mutual sharing of individual experiences, views and beliefs. 

If CA innovation was one part the equation, another equally important factor was the shift in power relationships between the academic team and CAs that occurred courtesy of project leader-/researcher-responsiveness. Initially, CAs were recruited to lead enquiry and generate information under the direction of the project leader. However, this relationship shifted, as CAs became increasingly recognised by the academic team as collaborators in reshaping the research process. In their reflections on challenges to co-produced research, Beebeejaun et al. [32] discuss how the priorities of the researchers may differ from those of the communities being researched. In this project, the priorities of participating community members differed from those of the academic team. However, the project leader’s amenability to change created a democratising space for discussion about rethinking the project approach. For academic researchers, this is not a comfortable space to occupy in a competitive funding environment where research outcomes and outputs need to be stipulated and delivered and where value for money also needs to be demonstrable. 

This turning point also relied heavily on the reflexive capacity of the project leader to be attuned to the perspectives of community members who were closer to the research field than the academic team. Further, the shift in power relations required researchers and CAs to live with uncertainty and ambiguity as a shared position that, combined with reflexive leadership, can provide a catalyst for developing more responsive approaches to co-produced research. Specifically, CAs had to live with uncertainty about the assessment tool and the frustrations of making initial contact with managers. Thomas-Hughes [25] discusses the importance of critically reflecting on and recognising the “messiness” of co-produced research to identify the ethical tensions, challenges and contestations of power that can emerge during the process. While being immersed in the research process can generate feelings of frustration and anxiety, in this case such immersion provided opportunities for taking stock of aims and progress and reconfiguring new directions. 

The project achieved change at a micro-level; this is evident in the small but notable shifts noticed by CAs in staff responses to the life-stories of LGBT individuals. Similar shifts were discernible in interview accounts from managers pre- and post-intervention, who started to consider and, in some cases, initiate their own strategies for organizational change. The relationships developed between CAs and care home staff and managers were critical in facilitating these changes and we are uncertain as to whether these relationships would have emerged without the advisory sessions. The project did not achieve changes in the overall climate of participating care homes (or at least the evaluation methodology did not capture this) but this goal was too ambitious anyway within the context of a short-term project. The approach used has potential to advance a social inclusion agenda in residential care homes but, again, that would be contingent on time-scale and ongoing collaborative relationships with community members. The project was a staging-post in what needs to be a longer-term campaign at a national level. 

Scaling up this project would take time and financial resources. Initially, replication of the project might be a feasible first step—meaning to implement the learning from this project across other care home settings. The WHO [47] recommends the development of strategies to assist this by:

… disseminat(ing) knowledge about the innovation … to stimulate motivation and the willingness to implement of eventual and potential user organizations

*(p. 41)*

We have achieved a great deal of interest via our dissemination activities including attracting interest from national bodies such as the Care Quality Commission (CQC) and the Social Care Institute for Excellence (SCIE). The WHO (2016) report identifies important facilitative aspects to scaling up including the personal commitment of the project partners and recognizable benefit for the population. However, a key challenge remains which relates to financial issues and to the amount of administrative work.

Central to rapport-building with staff was CAs willingness to share their own life-stories. In various ways, the biographies of academic team members and CAs were enmeshed in the research process. As team members, we brought a commitment to initiating and facilitating a process of change to improve care conditions for older LGBT residents—a subject position of being “cared for” that we may occupy at some point in our lives. For CAs, this involved telling their own life-stories of “coming out” and identifying as LGBT. At some points, this was a vulnerable position to occupy and required countering in the moment prejudiced responses from staff. 

While some commentators have promoted the benefits of autobiographical and dialogical approaches to enhancing the awareness of health and social professionals towards LGBT identities and life-stories [48,49], less attention has been given to the welfare of people sharing their personal stories and accounts. Other commentators in social psychology have highlighted the ways in which LGBT citizens can experience vicarious stressors across social environments in which heterosexist views and beliefs are transmitted [50,51]. Researchers and educators adopting this approach across other health and social care projects must ensure that rapid support can be provided to community members working in risky environments offering their own accounts and being “out” to staff. This includes preparatory activities to determine if community members are sufficiently equipped and supported to manage hostile responses from participants. Social support is recognised as a resilience factor for LGBT individuals, due to its association with the ability to process negative responses [52]. The project leader was key in providing regular one-to-one support (telephone debriefs; meeting in person) as well as facilitating the CAs meeting together for providing peer support. 

This is as much an ethical requirement as it is a strategy for sustainability. Crawley and Broad [53] caution that inviting queer speakers to share LGBT life-stories with unfamiliar audiences can reiterate formulaic “coming out” stories and neglect to convey the heterogeneity of individual life-experiences. This limitation was partly evident in our project as CAs conveyed life-stories from primarily lesbian, gay, and trans standpoints and input on experiences of intersex and bisexual individuals was marginal. This is a further consideration for qualitative researchers seeking to emulate this approach across other organizations and services. 

## 6. Conclusions

This article has distilled reflections on an action-oriented research process that was founded on principles of collaboration with community advisors and the participating provider and committed to identifying strategies for improving the responsiveness of care home managers and staff to LGBT residents and their significant others. It was designed to contribute to the inclusion of older LGBT residents—inclusion being the key to supporting and maintaining positive health and well-being in a risk environment that houses a seldom-heard social group. In particular, we have discussed the conditions that enabled co-production to emerge as an unintended but powerful process for initiating small-scale change in care home environments for older people. 

Our reflections on this process convey a number of takeaway messages for social researchers and workplace teams investing in a similar model of organizational enquiry and change. Enabling a co-production turn relies on: reflexive leadership; receptiveness to change in direction and approach to enquiry; willingness to live with a productive uncertainty/ambiguity; and openness to shifting power relationships between research teams and stakeholders. Our reflections also underscore the importance of LGBT storytelling as a non-didactic strategy for fostering understanding and building relationships between stakeholder groups in non-judgmental spaces where staff and stakeholders can explore the workings of sexuality-based prejudice and discrimination. This method has transferability across health and social care services and organizations. 

## Figures and Tables

**Table 1:** Intended Project Activities versus Actual Project Activities led by the Project Leader.

Planned Project Activities	Actual and Revised Project Activities
1 Academic team develop assessment and audit tool from research evidence for service audit	1 Academic team develop assessment and development tool from research evidence for service audit
2 Recruit Community Advisors through participating provider‘s volunteer recruitment process	2 Recruit Community Advisors through participating provider‘s volunteer recruitment process
3 Community Advisors training programme (1.5 days)	3 Community Advisors training programme (1.5 days)
4 Community Advisors to conduct audit with care home managers	4 Community Advisors informal meetings with managers and staff
5 Team report back, review and action planning	6 Community Advisors develop formal “advisory” sessions for care home staff—facilitated by the project leader
6 Develop and implement local action plan in each of the six care homes	7 Regular debriefing one-to-one with Community Advisors by project leader (telephone, face-to-face, on-site)
	8 Team report back and review “audit process” to accommodate revised “starting blocks”
	9 Development of care home champions scheme working closely with Community Advisors
	10 Regular review meetings led by Community Advisors and the project leader (three meetings).
	11 Care home champions conduct assessment and development audit guided by regular input by from Community Advisors
	12 Action plans developed across the six care homes with leadership of particular areas based on strengths of each individual care home
	13 Action plan adopted by care homes in conjunction with participant provider‘s LGBT Staff and Residents Advisory Group.
